# Signal Peptides: From Molecular Mechanisms to Applications in Protein and Vaccine Engineering

**DOI:** 10.3390/biom15060897

**Published:** 2025-06-18

**Authors:** Shuai Zhang, Zhihui He, Hui Wang, Jingbo Zhai

**Affiliations:** 1 School of Basic Medical Sciences, Inner Mongolia Minzu University, Tongliao 028000, China; adonis98_6517@163.com (S.Z.); zh0475@163.com (Z.H.); pluto_0223@163.com (H.W.); 2 Key Laboratory of Zoonose Prevention and Control at Universities of Inner Mongolia Autonomous Region, Tongliao 028000, China; 3 Brucellosis Prevention and Treatment Engineering Research Center of Inner Mongolia Autonomous Region, Tongliao 028000, China

**Keywords:** signal peptide, eukaryote, protein, transmembrane transportation, amino acid

## Abstract

Signal peptides (SPs) are short amino acid sequences located at the N-terminus of nascent proteins and are widely present across various life forms. They play crucial roles in protein synthesis, transmembrane transport, and intracellular signal transduction. With the rapid advancement of bioinformatics, studies have revealed that the functions of SPs are far more complex than previously understood. In recombinant protein expression systems, the rational design and optimization of SPs are essential for enhancing the expression efficiency and secretion level of exogenous proteins. Meanwhile, the application value of SPs in vaccine development has attracted increasing attention. This review summarizes the structural characteristics, functional mechanisms, and applications of SPs in recombinant protein production and SP-based vaccines. It also discusses their biological roles, the significance of engineering optimization strategies, and the current challenges, aiming to provide theoretical support and practical guidance for improving recombinant protein yield and advancing SP-based vaccine development.

## 1. Introduction

The transmembrane transport of proteins is a fundamental process that sustains normal cellular and biological activities and is an essential function of the cell membrane. In both eukaryotic and prokaryotic organisms, most secretory proteins possess a short peptide chain of 16–30 amino acid residues at their N-terminus, known as a signal peptide (SP) [1]. Additionally, some SPs are found within organisms, located at the C-terminus or internally, with structures differing from conventional SPs [2].

In 1971, Blobel et al. first proposed the hypothesis that specific segments of amino acid sequences could influence protein transport and localization [3]. In 1972, Milstein et al. reported that after the maturation of IgG light chain precursors within the endoplasmic reticulum (ER) vesicles, mature IgG was found to lack 20 hydrophobic amino acids at its N-terminus [4]. Building on this, Blobel et al. proposed the “Signal Hypothesis” in 1975, suggesting that the N-terminus of newly synthesized secretory polypeptides contains a sequence that directs the nascent polypeptide to be recognized and bound by receptors on the rough endoplasmic reticulum (RER) membrane. The SP traverses a membrane channel into the ER lumen, where it reaches a specific stage before being hydrolyzed by signal peptidase (SPase) at a cleavage site on the SP. The cleaved polypeptide chain then undergoes folding, glycosylation, and other modifications to ultimately form a functional secretory protein [5]. The “Signal Hypothesis” provided a clear explanation for the key mechanism by which secretory proteins are synthesized on free cytoplasmic ribosomes and subsequently directed into the ER, thereby filling a crucial gap in our understanding of early intracellular protein secretion processes. Between 1978 and 1980, further studies identified key components of the membrane insertion and translocation mechanisms, including signal recognition particles (SRPs), SRP receptors, and the protein translocation channel [6]. Subsequently, Blobel expanded the “Signal Hypothesis” into the “Topological Hypothesis”, emphasizing that multiple sequences (such as leader sequence and sorting sequence) may act synergistically in intracellular protein targeting and localization, collectively shaping the topological structure of intracellular proteins [7]. In 1987, Deshaies et al. discovered the sec61 mutant complex in yeast, which exhibited defects that caused various secretory proteins to accumulate within the cell during early secretion stages, leading to transport blockage. Sec61 was later confirmed to be a key component of the protein translocation channel [8,9]. In 1993, Rapoport et al. successfully reconstituted the process of protein transport and membrane integration into mammalian ER membranes by incorporating sec61 and SRPs into liposomes [10]. This translocation process was found to occur without the involvement of the Recombinant Translocation-Associated Membrane Protein 1 (TRAM1), simplifying the mechanism. In 1999, Blobel was awarded the Nobel Prize in Physiology or Medicine for the “Signal Hypothesis”, acknowledging his discovery that proteins possess intrinsic signals that regulate their intracellular transport and localization. This review provides an in-depth discussion of SPs, elaborates on their structure and function, introduces their applications, and explores future directions, aiming to offer theoretical and practical insights for related research.

## 2. SP Structure

**Figure 1:**
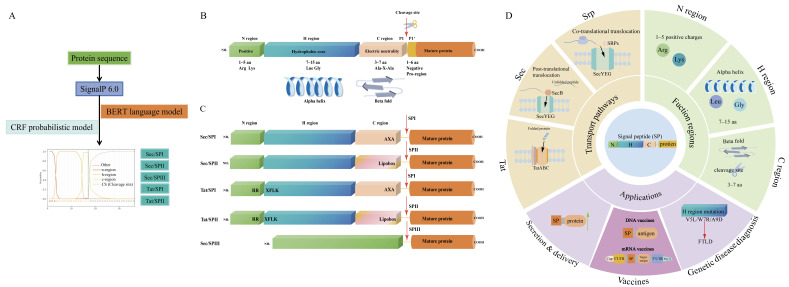
A comprehensive analysis of SP prediction, structure, and application. (**A**) The workflow of SP prediction using SignalP 6.0. The input sequence is encoded by BERT LMs and analyzed by a CRF probabilistic model to identify the SP type and cleavage site. (**B**) A schematic diagram of the typical structure of the SP. The basic structure is divided into three regions, namely, the N-region (positively charged), the H-region (α-helix), and the C-region (β-fold). The region after the C-region and before the mature protein is called the pro-region. (**C**) The structures of different types of SPs. According to different transport pathways and the cleavage characteristics of SPase, SPs are classified into five types (Sec/SPI, Sec/SPII, Sec/SPIII, Tat/SPI, and Tat/SPII). (**D**) The protein translocation pathways mediated by SPs and their related application. SPs with different hydrophobicities are directed to distinct translocation pathways. SPs have multiple applications, including protein production and delivery, vaccine development, and genetic disease screening.

SignalP 6.0 (Version 6.0, DTU Health Tech, Lyngby, Denmark) is a machine learning model that combines bidirectional encoder representations from transformer (BERT) language models (LMs) with conditional random fields (CRFs) designed for SP prediction (Figure 1A). An SP consists of three main regions—the N-region, H-region, and C-region—collectively forming the classic N-H-C structure (Figure 1B). They play a crucial role in guiding the localization and transport of nascent proteins [11]. Typically, SPs are 15–30 amino acids in length and are recognized and cleaved by type I signal peptidase (SPI). Although classical SPs share a common structural framework, their amino acid composition varies across different organisms and target proteins. Notably, some unusually long SPs have been identified, such as the feline immunodeficiency virus (FIV) envelope glycoprotein (Env), which extends up to 175 residues [12]. Across viral, prokaryotic, and eukaryotic systems, certain long SPs resist cleavage post-translocation, endowing their protein with additional functions, such as immune response regulation and stability enhancement [13,14,15]. Meanwhile, some specialized SPs are considerably shorter. Twin-arginine translocation (Tat) SPs are shorter, averaging 14 residues, and are primarily located in the N-region [16].

According to SignalP 6.0, SPs are classified into five types based on their transport mechanisms and SPase specificity (Figure 1C): Sec/SPI, Sec/SPII (lipoprotein Sec-type), Tat/SPI, Tat/SPII (lipoprotein Tat-type), and Sec/SPIII (non-classical Sec-type) [17]. Lipoprotein SPs (Sec/SPII and Tat/SPII) are characterized by a conserved lipobox motif (Leu-(Ala/Ser)-(Gly/Ala)-Cys) in the C-region [18]. This motif facilitates the anchoring of bacterial lipoproteins to membranes through cysteine lipidation. Tat SP contains an RRXFLK motif in the N-region, whereas Sec/SPIII peptides, primarily found in bacterial type IV pilin precursors, lack a typical H-region [19]. SPs are sorted into three major translocation pathways (Srp, Sec, and Tat pathways) (Figure 1C). Functionally, they are widely applied in protein production, vaccine development, and the screening of genetic disorders (Figure 1D). The functional applications of SPs will be further discussed in the following sections.

Overall, while most classical SPs adhere to a well-defined structure and length, variations such as long or atypical SPs have evolved to meet the functional demands of specific biological systems, underscoring their diverse roles in cellular processes.

### 2.1. N-Region

The N-region of SP is typically composed of short, positively charged basic amino acids such as lysine (Lys), arginine (Arg), and histidine (His). This region primarily interacts with the phospholipid bilayer, SRPs, and the Sec translocation pathway, facilitating the translocation of nascent proteins. In both bacteria and eukaryotes, methionine (Met) is often the first amino acid in an SP, typically associating with tRNA on the ribosome to bind to the start codon (AUG) on mRNA, thereby initiating the translation of the nascent protein [20,21]. In bacteria, Met undergoes formylation to produce formyl-methionine (fMet), which then initiates polypeptide chain synthesis [22]. In eukaryotes, the initial Met remains unmodified and directly participates in translation [20,23]. The typical N-region sequence follows Met-Arg/Lys-X, with its length varying among different types of SPs. Tat SPs have a longer N-region than Sec SPs [24]. Among organisms, eukaryotic SP N-regions are generally shorter than those in prokaryotes, while Gram-positive bacteria have longer N-regions than Gram-negative bacteria [25].

#### 2.1.1. The Role of Positive Charges in the N-Region

The positive charges on the N-region, along with the H- and C-regions, preserve the correct topological structure of the polypeptide during its translocation process [26]. Positive charges in SPs play a crucial role in the secretion of small eukaryotic proteins. As the SP guides the synthesis of the nascent peptide chain, electrostatic interactions between the peptide and the negatively charged membrane allow the chain to enter the membrane. The SP is cleaved by SPase I, and it remains on the inner side of the plasma membrane [27].

#### 2.1.2. The Influence of Positive Charges in the N-Region on Translocation Efficiency

Studies indicate that the removal of basic residues from the N-region adversely affects both the translocation efficiency and the synthesis rate of nascent proteins [28]. The net positive charge and its distribution in the N-region directly influence the entire translocation process of the SP. Generally, eukaryotic SPs have a lower net charge compared to bacteria, with Gram-positive bacteria exhibiting a slightly higher net charge than Gram-negative bacteria; both types yield mature peptides that are negatively charged [29,30]. In common expression systems like *E. coli*, maltose-binding protein (MBP), with a net charge of +1, exhibits the highest translocation efficiency [25]. Increasing the net positive charge of the *B. subtilis* SP N-region improves the processing efficiency of the SP [31,32].

Research has shown that point mutations in the N-region of the chimeric mossy hydrolase (mHG) lead to a significant decrease in mHG secretion when the N-region carries four positive charges [33]. This phenomenon may be attributed to tighter binding between mHG and SRPs, hindering the release of the enzyme precursor. In a saturation mutagenesis study of the *B. subtilis* α-amylase (AmyE) SP N-region, reducing three positive charges to two led to more than a threefold increase in protein secretion activity [28,34]. Therefore, the relationship between net charge and translocation efficiency is not simply linear. Merely increasing the number of positive charges in the N-region is not always the optimal strategy.

When the net charge is zero or negative, it may slow down the protein’s translocation to the extracellular space [35]. In some cases, changes in the charge number might have a minimal impact on protein synthesis. In the mitochondrial protein translocation system, the matrix-targeting sequence (MTS) is a special SP with positively charged basic amino acids at the N-terminus, facilitating the translocation of precursor proteins across membranes [36]. Researchers replaced three key basic amino acids in the MTS sequence with electrically neutral Ala to construct the Oxa1-AAA-GFP mutant. The results showed no difference in the expression level between the mutant and the wild type, suggesting that the amino acid replacement did not induce protein degradation or misfolding [37]. However, the mitochondrial import rate of the mutant was delayed, demonstrating that these positively charged basic amino acids are crucial in regulating the rate at which the Oxa1 protein enters mitochondria.

The N-region plays a crucial role in optimizing SPs. While individual positively charged amino acids contribute to creating a positively charged environment, the functionality of the N-region depends on the specific arrangement of these amino acids, not just a single residue. Generally, a net positive charge of two or three is sufficient for modification requirements. The optimal net charge, however, may vary depending on factors such as the precise sequence of the SP, the type of protein being targeted, and the cellular environment.

### 2.2. H-Region

The H-region represents the hydrophobic core functional region of the SP, which can form an α-helix conformation. Its major function is to assist in targeting, embedding, and translocating the nascent peptide across the cell membrane during the transport process. This region ensures the correct association of the SP with the membrane and selects the appropriate secretion pathway (Srp, Sec, or Tat) for translocation based on hydrophobic preferences while also participating in the folding and glycosylation modification of the nascent peptide chain. The H-region typically consists of 7 to 15 hydrophobic amino acids, primarily composed of leucine (Leu), isoleucine (Ile), and valine (Val) [38]. Among these, Leu is the most frequently observed in the H-region, often exhibiting a conserved motif of three consecutive leucine residues (Tri-Leu), particularly in higher eukaryotes such as mammals. Studies have identified a highly conserved YSIRK/G-XX-S motif at the beginning of the H-region in Gram-positive bacterial cell wall-anchored surface proteins [39]. This motif directs the precursor secretory protein to localize specifically in the cell membrane region, demonstrating unique spatial localization and regulatory functions. Certain *S. aureus* secretory precursors contain this motif, and proteins that carry it coordinate the synthesis of lipoteichoic acid, thereby affecting the assembly efficiency of bacterial virulence factors [40]. This regulatory mechanism may represent a common feature in Gram-positive bacteria, though further verification is needed.

#### 2.2.1. Hydrophobicity and Transport Efficiency of the H-Region

The hydrophobicity of amino acids in the H-region is associated with transport efficiency. Research has shown that when glycine (Gly) at position 25 of the H-region in *E. coli* Tat-dependent SP TorA was mutated and replaced with Met, which is more hydrophobic (G25M), the transport efficiency increased compared to alanine (G25A). Conversely, in mutations to more hydrophilic amino acids, such as threonine (Thr) or asparagine (Asn), transport abnormalities occurred [41]. However, the relationship between hydrophobicity and transport efficiency is not always linear. Once the hydrophobicity exceeds a certain threshold, transport becomes increasingly challenging. Similarly, increasing the number of hydrophobic amino acids, altering its folding conformation, or introducing aromatic amino acids does not necessarily enhance transport efficiency. An H-region consisting solely of Leu showed the highest transport efficiency when containing eight consecutive Leu. The transport capacity diminished with 5 or more than 12 Leu [42]. In a system with alternating Leu and Ala, transport efficiency peaked at 10 alternating residues, whereas a sequence with 10 consecutive Leu showed almost no activity [42]. During optimization of the biosynthesis of the targeted anticancer drug bevacizumab, three-point mutations (L5W, L8V, and L13A) were introduced into the H-region, resulting in decreased antibody production [43]. Furthermore, the position of hydrophobic amino acid residues in the H-region of *E. coli* influences transport efficiency. In general, residues positioned closer to the N-region tend to show lower efficiency, while those positioned at the H-region center or near the C-region show relatively higher efficiency [44]. These findings suggest that the functionality of the hydrophobic region depends not only on the amino acid composition but also on the arrangement of amino acids and their secondary structure.

#### 2.2.2. Hydrophobicity and Transport Pathway of the H-Region

The transport pathway guided by the SP also varies depending on its hydrophobicity (Figure 1D). In general, highly hydrophobic SPs prefer the Srp pathway, while those with moderate hydrophobicity can be transported via both the Tat and Sec pathways, with Tat being the least hydrophobic of the two [44,45,46]. The weak hydrophobicity of Tat facilitates preventing misrecognition by SRPs, ensuring correct transport through the Tat pathway. In prokaryotes, both the Tat and Sec pathways coexist. Rieske iron–sulfur proteins in actinobacteria are unique, as their transmembrane domains contain homologous Tat SPs [47]. These proteins exhibit a Sec-Tat cooperative transport feature, where the Sec pathway alone is insufficient and requires Tat to complete transport.

#### 2.2.3. H-Region α-Helix and Transport Efficiency

The H-region of nascent peptides typically exhibits a characteristic α-helix secondary structure. This conformation interacts with key components of the secretion pathway, facilitating the efficient transport of cytosolic proteins into the periplasmic space [38,44]. Alterations in the α-helix conformation can lead to abnormal protein transport. Proline (Pro), Gly, and Ser are known as “helix breakers” because they disrupt the α-helix conformation and arrangement rule. This phenomenon typically does not occur at the edges of the H-region to avoid disrupting the continuity of the hydrophobic core [44,48,49]. L-asparaginase (L-ASP), replacing Gly with Leu, resulted in a more stable α-helix, but the secretion levels decreased [50]. Excessive stabilization of the α-helix conformation suppresses secretion efficiency. During the folding and glycosylation processes of HIV-1 Env’s gp160 SP in the ER, delayed cleavage occurs [51]. Introducing the M26P mutation alters the secondary structure, separating the H-region from the C-region and enhancing cleavage efficiency [51]. The gp160 SP overlaps with the C-region, with the α-helix extending to the cleavage site, resulting in steric hindrance that prevents the SPase from approaching and inhibits co-translational cleavage. Consequently, this prevents premature cleavage, ensures correct folding and functionality of gp160, and ultimately affects the virus’s adaptability and infectivity.

#### 2.2.4. H-Region Mutations in Hereditary Diseases

When the H-region is shortened, deleted during in vitro modifications, or mutated, it may lose its normal function, potentially inhibiting protein synthesis and leading to pathogenic mutations. Hemophilia B is characterized by a deficiency in coagulation factor IX (FIX). Mutations in the H-region of the FIX SP can lead to a significant reduction in FIX secretion [52]. This abnormal genetic mutation disrupts co-translational transport, causing the FIX SP to accumulate in the cytoplasm. The defective SP is degraded via the ubiquitin–proteasome pathway rather than through the regulation of aberrant protein production (RAPP) pathway [52]. When the SP fails to function properly, the cell tends to directly degrade the faulty protein rather than compensating through other regulatory mechanisms. Mutations in the H-region of SPs (V5L/W7R/A9D) have also been observed in frontotemporal lobar degeneration (FTLD) [53,54,55]. H-region mutations may also serve as potential early diagnostic markers for genetic screening in certain protein secretion disorders.

When optimizing the H-region, it is important to not only design hydrophobic amino acids carefully but also to consider the interactions between the N, H, and C regions to ensure synergistic effects. Increasing the positive charge in the N-region, enhancing hydrophobicity in the H-region, and minimizing polar amino acids in the C-region may help optimize the positioning of the H-region. An optimal H-region typically contains around five hydrophobic residues, achieving good transport efficiency while avoiding excessive hydrophobicity that could lead to protein aggregation or increased membrane stress. Modifications to the H-region should focus on its central region, but these design principles may vary depending on the specific context and system.

### 2.3. C-Region

The C-region, also known as the processing region, is located at the C-terminus of the SP. It contains a weakly conserved cleavage site recognized by SPase, allowing for precise cleavage of the SP at a specific site. The C-region cleavage site serves as a reference point, with upstream positions designated as P1 (−1) and P2 (−2) and downstream positions as P’1 (+1) and P’2 (+2). The cleavage step is crucial for the subsequent formation and functional activation of the nascent protein. The C-region typically consists of 3–7 neutral or polar amino acids [38,56]. These amino acids have hydrophilic side chains, which interact via non-covalent forces to stabilize the secondary structure. The length, cleavage site position, and conservation of the C-region can vary among different secretory proteins. The C-region of eukaryotic organisms is shorter than that of prokaryotes. In Gram-positive bacteria, the C-region is longer, comprising 9–11 residues, while Gram-negative bacteria usually have 5–6 residues [44,57]. The length of the C-region significantly impacts the processing efficiency of the nascent protein. When the C-region of *B. stearothermophilus* amylase was modified, the processing rate was highest with five amino acid residues. As the length increased, the processing efficiency decreased, and at 13 residues, processing was completely halted [57,58].

#### 2.3.1. Cleavage Motifs and Amino Acid Preferences in the C-Region

The C-region, as a substrate for SPase, follows the (−3, −1) rule, with cleavage occurring at specific positions (P3 and P1) [59,60]. A consensus motif exists before the cleavage site in the C-region. At the P1, Ala is the predominant amino acid, although other neutral amino acids may also be present in eukaryotic organisms [61,62]. Traditionally, the consensus motif for the C-region of SPs in organisms is Ala-X-Ala. However, studies have shown that there is variability in the cleavage motifs in prokaryotes. In most *B. subtilis* SPs, the motif is Val-X-Ala, where Ala is replaced by Ser, Leu, Ile, or Val, especially at the P3 position, providing more possibilities for amino acids [63]. In the *Alpha-proteobacteria* class, *P. ubique* has Ser at the P3 position (Ser-X-Ala), and in *E. chaffeensis*, both the P1 and P3 positions are entirely substituted with Ser (Ser-X-Ser). In the *S. phylum*, *L. interrogans* and *B. burgdorferi* display Leu and Ile at the P1 position, while the P3 position can be either Ala or Ser (Ala/Ser-X-Leu/Ile). Additionally, *M. hungatei* replaces P3 with Val (Val-X-Ala) [64]. The preferences for amino acids at the P1 and P3 positions are evident, whereas P2 seems to lack a clear dominant amino acid preference, exhibiting a degree of flexibility.

#### 2.3.2. The Role of the Pro-Region in Protein Secretion

A short peptide known as the pro-region (also termed the export initiation domain) is located upstream of the C-region cleavage site, preceding the mature protein (Figure 1B). This region has one to six residues from the N-terminus of the secretory protein but can extend up to 30 residues [16,65]. It is generally composed of neutral or acidic amino acids. The absence of this region can result in protein accumulation within the cytoplasm and forming inclusion bodies. Amino acid preferences also exist after the cleavage site, which regulate the cleavage efficiency. When Lys at P1’ of MBP was replaced with Pro, the mutant exhibited increased levels of unprocessed precursor protein, thereby disrupting the processing and maturation of the protein [30,66]. This indicated that introducing Pro at P1’ caused incompatibility, which inhibited cell growth and physiological function while further suppressing SPase cleavage activity. P2’ also exhibits a preference for negatively charged amino acids, with variations observed across the different proteins, Glu and Asn, while generally excluding aromatic amino acids [30,66]. Additionally, changes in cleavage efficiency are influenced by the cooperative effect of multiple sites. When constructing the *E. coli* TasT-MBP-P19/P29 fusion protein, it was found that the TasT protein could not be effectively cleaved by LepB. The introduction of P2’ Phe not only suppressed LepB cleavage activity, but amino acid residues at other cleavage sites (P3–P4’) also exhibited an inhibitory effect [66]. Point mutations in the C-region may alter the cleavage site. When Ser in the C-region of the SARS-CoV-2 spike (S) protein SP was replaced with Ile, the cleavage site shifted, which consequently improved the secretion capacity of the receptor-binding domain (RBD) protein [67]. Fusing the tissue plasminogen activator (tPA) SP with the RBD encoding sequence not only improved antigen secretion efficiency but also induced higher levels of IgG antibody titers and the Th1-type cellular immune response by extending antigen exposure time [68].

Overall, the pro-region is essential for efficient secretion, proper folding, and recognition by SPase I. However, the current understanding of its function remains limited, especially regarding its cooperative interactions with residues beyond the immediate cleavage site. Cleavage efficiency is not solely determined by individual residues but results from a complex interplay among multiple amino acids. Notably, the P1′ site plays a particularly critical role. Unfavorable substitutions (e.g., Pro) can introduce steric hindrance, thereby reducing processing efficiency. To prevent secretion failure or misprocessing, the charge distribution, structural flexibility, and membrane-specific context of the pro-region should be carefully considered.

#### 2.3.3. SPVs and C-Region Cleavage Errors

Cleavage errors at the cleavage site can lead to shortened or extended terminal sequences, which are commonly seen in recombinant protein production. The miscleaved SPs are referred to as signal peptide variants (SPVs). During recombinant protein production, the appearance of SPVs can potentially impact drug quality, safety, and efficacy, including structural–functional interference, post-translational modification abnormalities, and immunogenic risks [69]. The potential impact of SPVs has been further validated in studies of congenital antithrombin (AT) [70]. Although the AT SPV (C32W) mutation does not block transport, it modifies the cleavage site, resulting in the abnormal retention of unprocessed SPs and ultimately causing type I AT deficiency. Therefore, it is essential to enhance variant monitoring to improve the quality control of the recombinant protein.

When optimizing the C-region, it is preferable to follow the consensus motif. A C-region consisting of about five amino acids is optimal, with a general preference for neutral or weakly negatively charged residues to promote efficient cleavage by SPase. This region should have minimal steric hindrance to avoid unnecessary folding. By selecting appropriate amino acids based on the secretion pathway of the target protein and the amino acid preferences, the secretion efficiency of the target protein can be significantly enhanced.

## 3. SP Transport Pathways

**Figure 2:**
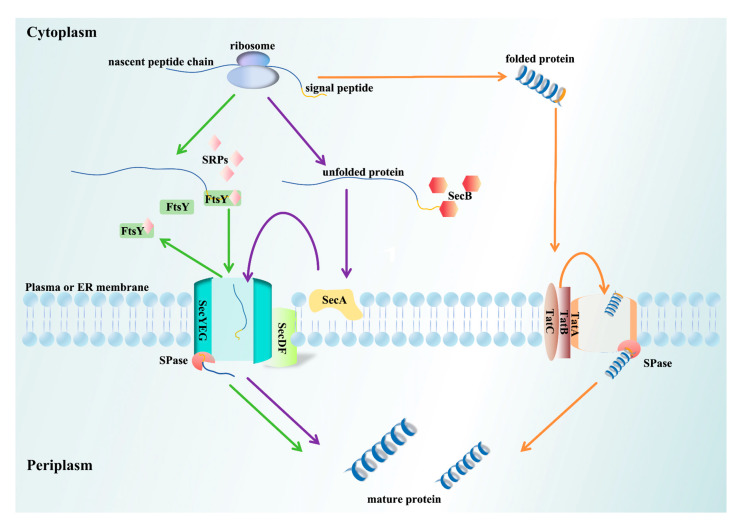
Schematic diagrams of different transport pathways. In the Srp pathway, co-translational targeting is mediated by the signal recognition particles (SRPs) and their receptor (FtsY). In the Sec pathway, the post-translational translocation of unfolded proteins involves SecB, SecA, and the SecYEG translocon. The Tat pathway translocates fully folded proteins via the TatABC complex. In bacteria, the translocation channel is located in the plasma membrane, while in mammals, it is positioned on the ER membrane. Green arrows: the Srp pathway; purple arrows: the Sec pathway; orange arrows: the Tat pathway.

The characteristics of classic SPs affect the pathways mediating protein transport, which are generally classified into co-translational transport (Srp) and post-translational transport (Sec), while Tat operates independently of the two (Table 1). The classification of protein transport pathways ensures efficient and accurate protein transport in different physiological environments, meeting the demands of rapid growth and high secretion (Figure 2). The evolution of transport mechanisms is driven by the structural composition, charge distribution, and hydrophobic properties of the SP and protein precursor. The various secretion pathways are not isolated but instead work together to ensure the correct localization and transport of proteins. As mentioned earlier, the insertion of the transmembrane helix of iron–sulfur proteins initially relies on co-translational translation via the Sec pathway, with final transport completed by the Tat pathway [47].

### 3.1. Srp Pathway

The SRP pathway is a highly conserved, co-translational targeting transport system that synchronizes transport with protein translation, preventing the modification of proteins in the cytoplasm [71]. This pathway is widely present across various organisms and primarily targets the ER in eukaryotes or the plasma membrane in prokaryotes [44,72,73]. After the N-terminal SP of the ribosome-nascent chain complex is exposed, it binds to SRPs, triggering a pause in translation. Subsequently, SRPs interact with the SRP receptor (SRα/SRβ in eukaryotes, FtsY in prokaryotes), targeting the Sec61p or SecYEG translocon to deliver the nascent peptide chain into the transmembrane channel. Upon completion of protein transport, SRPs and the SRP receptor dissociate, allowing the next cycle of protein transport to begin [74]. This process is tightly regulated by the GTPase cycle: initially, the free SRPs and receptor domains each bind to GTP, which keeps them in a relatively loose and inactive state [75]. When the ribosome–nascent chain complex carrying the SP forms, the SRPs recognize it and induce a conformational change, which brings the SRPs closer to the SRP receptor domain, forming an unstable transient dimer [76]. As further conformational adjustments and nucleotide binding occur, the dimer gradually stabilizes. During this process, the GTPase hydrolyzes GTP. The complex then interacts with the Sec61/SecYEG translocon, accurately delivering the nascent chain to the target membrane position. After the transport period is complete, the SRPs and the receptor dissociate, and the GTPase domain refolds, preparing for the next round of protein transport.

The Srp pathway exhibits a high degree of conservation, with the GTPase activity of the SRP54-SRP RNA core being present in all forms of life, while other components show species-specific differences [77,78]. In eukaryotes, the mammalian SRP complex consists of 7SL RNA and six protein subunits (SRP9/14/19/54/68/72) [79]. SRP19/54/68/72 forms the S domain, responsible for recognizing the SP and interacting with the SRP receptor. The heterodimer formed by SRP9 and SRP14 can bind to the Alu domain and interfere with the binding of the elongation factor eEF1A, inducing translation arrest to ensure the spatiotemporal coordination of co-translational transport [80]. Notably, the chloroplast Srp uses post-translational transport and has evolved the CPSRP43/CPSRP54 complex to adapt to thylakoid membrane characteristics [80]. In prokaryotes, it consists of 4.5S RNA and SRP54 (Ffh) protein [73]. In comparison, the subunit domains of eukaryotic Srp are more refined. Archaea retain some of the simpler, more direct mechanisms of bacterial Srp while also exhibiting more complex regulatory features like those of eukaryotes.

### 3.2. Sec Pathway

The Sec pathway is responsible for translocating unfolded proteins across membranes from the cytoplasm and is classified as a post-translational transport [81]. The Sec system comprises SecA (ATPase), SecB (a molecular chaperone), and SecY/SecE/SecG, which together form a heterotrimeric membrane channel [82]. SecYEG, a heterotrimeric complex composed of SecY, SecE, and SecG, forms the transport channel, with SecY containing ten transmembrane helices. SecE and SecG anchor to the cell membrane, ensuring the stability of the channel and facilitating transmembrane transport. Additionally, SecDF enhances the efficiency of proton pump transport. This pathway is conserved across nearly all forms of life [83]. In *E. coli*, 90% of proteins utilize the SecB-SecA cooperative pathway, while some Gram-positive bacteria (such as *S. aureus* and *Listeria*) rely on SecA2, and most bacteria depend on SecB [84,85]. The SecYEG translocase mediates both co-translational and post-translational transport [86].

In the post-translational transport process, the molecular chaperone SecB binds to the nascent polypeptide chain, preventing premature folding and delivering the unfolded chain to SecA. SecA not only facilitates the transfer of the polypeptide to the SecYEG translocon but also provides the energy required for transport through its ATPase activity, which hydrolyzes ATP [86,87]. Furthermore, the proton motive force (PMF) further drives this process [88]. Prior to translocation to SecYEG, the signal sequence of SecB is cleaved by SPases. The newly secreted protein then completes its folding in the periplasm, and the mature protein is released by either SecD or the SecDF complex. Additionally, this pathway undergoes various post-translational modifications (PTMs) that fine-tune their functionality and influence the efficiency of protein processing. The main post-translational modifications (PTMs) of SPs, including glycosylation, phosphorylation, and acetylation, play a crucial role in the regulation of protein function.

### 3.3. Tat Pathway

The Tat pathway serves as an independent protein transport system for folded proteins, often requiring the assistance of cofactors for the transport process. This pathway is found in the cytoplasmic membranes of most bacteria, archaea, chloroplasts, and mitochondria [89]. The composition of the Tat pathway varies across organisms: Gram-negative bacteria possess a system composed of TatA/B/C proteins, while Gram-positive bacteria and archaea consist solely of TatA and TatC [90]. In plant mitochondria, the Tat pathway includes only TatB and TatC, where their structures are most stable and represent the minimal unit of the Tat system [91]. The N-terminal region of the TatA protein forms a transmembrane helix that provides membrane anchoring, while the C-terminal region contains an amphipathic helix with a charged, basic nature [92]. TatB is structurally like TatA but has a longer C-terminal than TatA. TatC, a membrane protein composed of six transmembrane helices, can form a precursor complex with TatB [93]. Many pathogenic bacteria rely on the Tat pathway to secrete virulence factors, thereby enhancing their pathogenicity. In animal models, the virulence of *P. aeruginosa* and *E. coli* O157:H7 depends on the Tat pathway [94,95,96]. Additionally, Tat can transport folded proteins in association with cofactors, delivering them to the extracellular space to exert their effects [24]. In symbiotic microorganisms, such as rhizobia, the Tat pathway is used to secrete specific proteins that contribute to nitrogen fixation, enhancing the survival of leguminous plants [97].

The SP at the N-terminus of the Tat pathway contains the S/T-RRXFLK motif, which interacts with the conserved region of TatC, triggering the transport process [96]. Recent studies have simplified this motif to ZRRXΦΦ (Z refers to a polar amino acid residue, R represents arginine, and Φ denotes a hydrophobic amino acid) [98]. The H- and C-regions of the SP are involved in the interaction between TatB and TatC [41]. The hydrophobicity of the H-region influences the ability of the protein to bind with the TatBC complex, while the basic amino acids in the C-region prevent the SP from directing the protein to the Sec pathway, ensuring its transport via the Tat pathway. Upon the binding of the TatBC complex to the SP, the assembly of the Tat transport machinery is triggered. Driven by the proton motive force (PMF), TatA aggregates with TatBC to form the translocation site [99]. When the protein’s C-terminus reaches the periplasm and interacts with the SPase, the SP is cleaved, and TatA dissociates. The TatABC complex disassembles, completing the transport process.

### 3.4. Uncanonical Transport Pathway

The pathways mentioned above all rely on SPs, with nascent proteins being secreted via vesicles and delivered to functional regions after passing through organelles such as the ER and Golgi apparatus. Studies have shown that not all proteins depend on the ER–Golgi apparatus (Table 2). In eukaryotes, some proteins lacking SPs undergo secretion through unconventional protein export/secretion (UPE/UPS) pathways, which can be further classified into vesicle-dependent and vesicle-independent pathways depending on whether vesicles are required [100]. These secreted proteins perform various unique extracellular functions through the UPS pathway, such as cell signaling, immune modulation, and lipid metabolism. The UPS is divided into four types: Type I (vesicle-independent), where secretion of SP-lacking proteins occurs directly across the plasma membrane into the extracellular space through lipid diffusion and membrane remodeling. Type II (vesicle-independent), lipidated proteins are transported across membranes via ATP-binding cassette (ABC) transporters, such as the secretion of α-mating factor (α-MF) in yeast [101]. There is an ongoing debate regarding whether Hsp70 follows the Type II pathway. When ABC transporter inhibitors such as DIDS and glibenclamide are used, the secretion of Hsp70 is inhibited [102]. However, due to the non-specificity of these inhibitors, this inhibition may be indirect, and further evidence is needed to validate the relationship. Type III (vesicle-dependent) is a more common unconventional transport pathway, where proteins require membrane vesicles as intermediates for membrane transport and secretion. Type IV (vesicle-dependent), also known as the Golgi-adjacent secretion pathway for leaderless proteins, transports proteins across membranes to the extracellular space. Studies have shown that the GTPases Rab1/Rab2A, located in the intermediate membranes of the ER–Golgi system, cooperate with TMED10 to regulate the transport of leaderless proteins [103]. These findings broaden the theoretical framework of secretion biology and provide new insights into the targeted regulation of disease-associated secretory proteins.

In summary, the current research remains largely descriptive and classificatory, lacking in-depth analysis of the dynamic regulation of these mechanisms across different cell types and under physiological or pathological conditions. Future studies should integrate single-cell analyses and gene editing technologies to systematically elucidate the spatial and temporal specificity of UPS and its interplay and competition with classical secretion pathways, thereby providing a more solid foundation for the development of targeted therapeutic interventions.

## 4. SP Applications

In recent years, along with the development of bioinformatics and artificial intelligence, research on SPs has expanded from basic studies on protein secretion mechanisms to advanced fields, such as protein engineering and vaccine design. SignalP is a specialized tool to predict SPs. The evolution from SignalP 4.0 to the deeply integrated machine learning model of SignalP 6.0 enables the precise identification of five types of SPs and secretory peptides and is applicable to metagenomic analysis [17]. By rationally designing SPs through the dimensions of prediction–structure–evolution, this approach achieves an organic integration of artificial intelligence and bioinformatics with life science research. Researchers can rationally design SPs for different expression systems, providing new strategies and directions for efficient protein secretion and vaccine antigen expression.

### 4.1. SPs in Prokaryotic Expression System

Prokaryotes, commonly used as protein expression hosts, are widely applied in food, pharmaceuticals, and industry due to their rapid growth, simple genetic background, low cultivation cost, and ease of purification [104]. *E. coli* and *B. subtilis* are the preferred choices for the industrial production of simple proteins. The periplasmic space of *E. coli* contains peptidyl-prolyl isomerase and disulfide bond isomerase (DsbA), which facilitate the correct formation of disulfide bonds and ensure proper protein folding [105]. *E. coli* secretory proteins are transported via both the Sec and Tat pathways. The SPs of *E. coli*, including OmpA, OmpF, and DsbA, as well as metabolic transport-regulated alkaline phosphatase (PhoA), MBP, and pectinase (PelB), are widely used for the production of recombinant proteins [98,106]. In contrast, *B. subtilis* is better suited for large-scale industrial production due to its lack of an outer membrane, which enables it to directly release secretory proteins into the culture medium. Studies have demonstrated that *B. subtilis* secretes various proteins via UPS [107,108]. Commonly used SPs include neutral protease and fructosyltransferase. In the chitosanase system of *B. subtilis*, CtH2 chitosanase (CH2CSN), a natural mutant, exhibits a significant secretion advantage. Compared to the wild-type CH1CSN, the CH2CSN-SP is missing six amino acid residues at the N-terminus [109]. However, the expression level of CH2CSN is 2.3 times higher than that of CH1CSN, and it is capable of secreting proteins into the extracellular space. Notably, the net positive charge of CH2CSN-SP decreases from +2 to +1 [109]. This observation challenges the assumption that increasing the positive charge universally enhances secretion efficiency. From another perspective, the N-terminal truncation observed under natural conditions may also represent an adaptive evolutionary strategy. Rather than being a neutral or deleterious mutation, this modification might have been positively selected to enhance the efficiency of protein secretion, possibly by improving structural compatibility between the SP and the host’s secretion machinery.

In another study, replacing the SP of *E. coli* Ag43 with PelB, PhoA, and OmpC/F SPs significantly improved the RBD display efficiency and loading capacity [110]. Additionally, temperature significantly influenced the display of neutral substrates for RBD [110]. At 20 °C, protein expression was higher than at 30 °C when using the above SPs, possibly due to enhanced protein stability at lower temperatures. Although dual SPs improved surface display efficiency in some fusion constructs, their overall performance was still inferior to that of single SPs. This difference is likely not due to a simple additive effect of using two SPs but rather the result of competition in the SP cleavage, insufficient coordination during translocation, and potential structural incompatibilities. These findings offer an important technological platform for the development of candidate vaccines. The aforementioned study significantly enhanced the surface display efficiency of the Ag43 system through SP engineering; however, several important areas remain to be explored. These include expanding the library of native SPs, validating the conformational integrity and functionality of prokaryotically expressed antigens, and addressing challenges such as protein degradation rates or folding states under low-temperature induction. These aspects are essential for advancing this technology from the laboratory toward clinical applications.

### 4.2. SPs in Eukaryotic Expression System

Eukaryotic expression systems mainly include yeast, insect, plant, and mammalian systems. Among these, the *P. pastoris* secretion system in yeast expression systems is advantageous for industrial-scale recombinant protein production due to its efficient secretion capacity and post-translational modifications. Common endogenous SPs used in this system include acid phosphatase Pho1, α-MF, sucrose invertase 2 (SUC2), and inulinase (INU) [100]. The existing pPICZαA and pPIC9K secretion expression vectors primarily employ the α-MF SP. Furthermore, the human serum albumin SP is frequently used for producing humanized proteins to reduce immunogenicity and improve biocompatibility [111]. The insect cell–baculovirus expression system (Sf9, High Five, and MultiBac cells) is a well-established eukaryotic system capable of post-translational modifications and expressing complex intracellular and viral proteins [112,113]. The expression system exhibits strong adaptability to SPs from various species, with commonly used peptides, including bee venom peptide and the glycoproteins GP64 and GP67, which significantly enhance secretion efficiency [114,115,116]. The structural protein GP64, a key protein of the Bombyx mori nucleopolyhedrovirus, possesses a unique SP retention mechanism, with its N-terminal domain containing an uncleaved SP. The recognition site for the GP64 SPase is embedded within an α-helix, which prevents efficient recognition by the SPase, maintaining the GP64 conformation and enhancing virus infectivity [13]. Although GP64 enhances secretion efficiency, its performance across diverse host systems and with a broader range of heterologous antigens remains to be fully validated. The plant expression system utilizes its genetic transformation capabilities to establish transient or stable recombinant protein platforms in plant cells or tissues, effectively avoiding contamination from animal viruses and other sources [13,117]. The tobacco transient expression system has the excellent advantages of high-efficiency protein secretion and rapid production cycles, making it an ideal platform for responding to public health emergencies in remote, resource-limited areas, such as the production of monoclonal antibodies, like ZMapp against the Ebola virus [118]. Plant small-molecule SPs, including extracellular post-translationally modified precursor peptides with N-terminal SPs shorter than 120 amino acids, participate in plant developmental processes [119]. Mammalian systems have relatively few characteristic SP elements compared to other systems. Chinese hamster ovary (CHO) cells and HEK293 (human embryonic kidney) cells are common tool cells for protein expression, offering a near-native protein state and providing the stable integration of exogenous proteins [120]. Frequently employed SPs include human IL-2, human serum albumin, mouse IgG1 kappa chain and heavy chain, basement membrane protein (BM-40), myeloid cell differentiation antigen (CD33), tPA, and influenza virus hemagglutinin (HA) [43,121,122,123,124,125].

Despite the advantages offered by eukaryotic expression systems, significant challenges remain, including the following: (a) Species-specific SPs: The same SP may perform very differently in yeast and mammalian systems, which limits the design and application of universal SPs. (b) A lack of comprehensive comparative studies: Few studies evaluate SP performance across diverse eukaryotic hosts and target proteins, restricting the broad application of the current findings. (c) Limited adaptability for industrial-scale production: Although SP optimization shows significant improvements in the laboratory, maintaining high efficiency and stable expression in large-scale production remains challenging. In the future, high-throughput screening can be employed to construct SP libraries tailored to specific host organisms. Systematic comparative studies should be conducted to evaluate the performance of SPs across different hosts and target proteins, aiming to identify common patterns and key influencing factors. By integrating machine learning models and synthetic biology, computational prediction and screening methods can be used to design artificial SPs with enhanced secretion efficiency and greater adaptability, addressing the diverse needs of protein expression and industrial-scale production.

### 4.3. SPs in Vaccine Enhancement

SPs guide nascent polypeptide chains into the ER or other organelles, while their structural domains also possess strong immunogenicity and are rich in CD8^+^ T-cell epitopes. This feature can significantly enhance antigen presentation efficiency and immune recognition [126]. SPs improve vaccine effectiveness by refining the delivery and display of antigens to the immune system. This enhancement can result in more robust immune responses and increased protection against diseases. SPs can function as “endogenous adjuvants” by optimizing antigen processing and presentation, in turn enhancing the specific T-cell response and strengthening immune protection.

#### 4.3.1. SPs in Enhancing Subunit Vaccine Immunogenicity

SPs are extensively utilized in recombinant vaccine development, including vaccines targeting the Human Immunodeficiency Virus Type 1 (HIV-1). In HIV-1 vaccines, the primary immunological target is the Env precursor protein gp160, which undergoes host cell-mediated cleavage into gp120 and gp41 subunits. These subunits assemble into functional trimers, critical for eliciting effective immune responses [127]. However, extensive glycosylation and high conformational plasticity of the Env protein hinder the generation of broadly neutralizing antibodies [128]. Notably, substantial variation exists in the N-terminal SPs of Env proteins across different HIV-1 isolates [129]. By swapping the SPs between an acute-phase strain (AA05) and a chronic-phase strain (AC02), researchers demonstrated that the AC02-derived SP could enhance the immunogenicity of the AA05-derived gp120 subunit vaccine, making its antigenic properties more similar to those of AC02 [130]. This finding suggests that SP replacement not only regulates antibody induction but also reshapes glycosylation site distribution on Env proteins, thereby exerting a “programming” effect on vaccine immunogenicity.

Subunit vaccines inherently exhibit lower immunogenicity compared to live-attenuated or virus-like particle vaccines [131]. Traditional strategies rely on exogenous adjuvants, such as alum, AS02, and MF59, to drive robust host immune responses [131,132,133,134]. Interestingly, SPs themselves can function as “endogenous adjuvants”. For instance, the SP of bacterial lipidated proteins is recognized by the TLR2 receptor, triggering intracellular signaling cascades that lead to the release of pro-inflammatory cytokines like TNF-α and IL-6 [135]. This activation promotes dendritic cell maturation, enhances antigen presentation, and stimulates T- and B-cell responses, thereby increasing Th1-biased cellular immunity and neutralizing antibody production. Furthermore, SPs help maintain immune homeostasis by regulating cytokine release and preventing excessive immune activation [135,136].

Together, these findings underscore the dual role of SPs in both optimizing antigen properties and modulating host immune responses, offering promising strategies for subunit vaccine development. However, as the current evidence is primarily derived from animal models, such as Balb/c mice, the extent to which these effects can be replicated in human systems remains unclear. Further studies are needed to assess their scalability, consistency, and translational potential in clinical settings.

#### 4.3.2. SPs in Enhancing DNA Vaccine Immunogenicity

The integration of SPs into DNA vaccine constructs has emerged as an effective strategy to enhance antigen expression, secretion, and immunogenicity within the host. By fusing the SP to the antigen-encoding gene, vaccines can promote the efficient delivery of antigens to appropriate cellular compartments, thereby facilitating antigen processing and presentation.

An advanced approach involves the incorporation of dual SPs to further optimize antigen trafficking. In the human papillomavirus type 16 (HPV16) E6/E7 DNA vaccine, researchers fused a calcium-binding protein SP (CRT-SP) at the N-terminus and an ER retention signal (KDEL) at the C-terminus of the modified E6/E7 antigen. In a BALb/c mouse model, the SP-E6E7m-KDEL chimeric vaccine successfully directed the antigen into the ER lumen, enhancing MHC I molecule presentation and activating CD8^+^ T cells [137]. Although this vaccine has not yet progressed to clinical trials and remains limited to animal studies, the strategy notably improved antitumor immunity while reducing cellular stress induced by high levels of protein expression, thus helping to maintain intracellular homeostasis. These findings lay a promising foundation for future vaccine development. SPs have also been utilized to improve livestock DNA vaccines. In a novel vaccine targeting somatostatin-14 (SS-14), the tPA SP was fused to the SS-14 antigen gene, while CpG motifs served as immune adjuvants. This vaccine enhanced serum levels of growth hormone and prolactin, ultimately improving milk production and quality in goats within a short period [138]. The combination of SPs and molecular adjuvants significantly strengthened the vaccine’s ability to stimulate antigen expression and immune activation while also indirectly regulating hormone secretion.

To date, there are no fully approved DNA vaccines incorporating SPs on the global market for humans. The only DNA vaccine granted emergency use authorization is ZyCoV-D, developed in 2021 (Zydus Lifesciences Ltd., Ahmedabad, India) against SARS-CoV-2 [139]. This vaccine encodes the viral spike (S) protein fused at the N-terminus with a human IgE SP to enhance antigen secretion and immunogenicity. In addition, delivery via a needle-free injection system (NFIS) further improved its immune response and protective efficacy against SARS-CoV-2 [140]. ZyCoV-D, as a prophylactic vaccine, demonstrated an efficacy of 66.6% in its phase III trial. However, the study had notable limitations, including a gender-biased cohort, absence of age-stratified efficacy data, and lack of routine clinical parameter assessments (e.g., hematological, hepatic, and renal functions). Additionally, the sample size and follow-up duration were limited. Despite these constraints, ZyCoV-D represents the first DNA vaccine incorporating an SP to show protective efficacy against SARS-CoV-2 in a large-scale clinical trial [141]. In addition, the ZIKV DNA vaccine (GLS-5700), which incorporates an IgE SP to enhance antigen expression, has completed a phase I clinical trial (NCT02809443). The study demonstrated the induction of neutralizing antibodies in over 60% of participants.

These results highlight the potential of SP-engineered DNA vaccines in combating emerging infectious diseases. This highlights the broad applicability and flexibility of DNA vaccine platforms incorporating SPs. However, the limitations of the vaccines should not be overlooked. One major concern is the potential long-term persistence of plasmid DNA after administration, which raises potential risks of insertional mutagenesis due to possible integration into the host genome. Therefore, long-term safety monitoring remains essential for the clinical translation of such platforms.

#### 4.3.3. SPs in Enhancing Viral Vector Vaccine Immunogenicity

In the field of viral vector vaccines, the incorporation of SPs into antigen constructs has been shown to further enhance immunogenicity. For instance, a recombinant simian adenovirus 36 (SAd36) encoding the multi-stage proteins of *Plasmodium falciparum* was engineered by adding a mouse-derived IgGκ light chain SP upstream of the antigen. In a mouse model, this SP-SAd36 vaccine demonstrated dual immunological enhancements compared to the unmodified vaccine, including increased antibody affinity and significantly elevated cytokine secretion, particularly IFN-γ and IL-2 [142]. Similarly, in the development of a recombinant adenoviral vector-based Herpes Zoster candidate vaccine (ChimAd-tPAgE), the introduction of a tPA SP significantly boosted the antigen-specific T-cell immune response [143].

Moreover, a novel adenovirus-based vaccine targeting *N. meningitidis* Group B (MenB) was constructed using an AdHu5 vector incorporating the tPA SP. This vaccine utilized a dual-component SP strategy, combining the N-terminal SP of the MenB factor H binding protein (fHbp) with a bacterial lipid–box motif (LTAC) to form a full-length signal sequence (FLSS) [144]. The FLSS design enhanced antigen expression, localization, and secretion efficiency within infected HeLa cells, leading to improved immunogenicity. Compared with truncated or point-mutated SPs, the FLSS construct enabled higher and more stable antigen expression early after infection [144]. In BALb/c mouse models, an FLSS-based MenB vaccine elicited more rapid and robust immune responses than the licensed 4CMenB, achieving protective SBA titers after a single dose, whereas 4CMenB required multiple doses to reach comparable levels. Using dual-component SP strategies, such as combining bacterial motifs with antigen-specific SPs, vaccines can achieve improved immunogenicity and more stable expression. These innovations offer promising strategies for optimizing viral vector-based vaccine development. The aforementioned SP-engineered viral vector vaccine studies remain at the preclinical stage. Further translational research is necessary to determine whether the immunological advantages observed in animal models can be replicated in humans, particularly concerning long-term efficacy, safety, and manufacturing feasibility.

Currently, Ad5-nCoV (Convidecia™), developed by CanSino Biologics (Tianjin, China), is a recombinant adenovirus type 5 vector vaccine expressing the S protein of SARS-CoV-2 [145]. It was approved in several countries (e.g., Morocco, Malaysia, Indonesia, and China) for protection against COVID-19 during the pandemic. The success of Ad5-nCoV demonstrates the potential of adenovirus vector platforms; however, the types and quality of immune responses it induces vary among different populations. In-depth studies on the maintenance mechanisms of immune memory are crucial for optimizing vaccine strategies.

#### 4.3.4. SPs in Enhancing mRNA Vaccine Immunogenicity

The introduction of SPs in mRNA vaccines has become a pivotal element in optimizing antigens. Currently, most mRNA vaccines commonly incorporate the tPA SP to enhance immunogenicity and protective efficacy [146,147,148,149]. Researchers have constructed three types of mRNA vaccines targeting the S protein: one with the full-length sequence (including the SP), one with a deletion of the transmembrane region and cytoplasmic tail, and one with both the SP and the transmembrane region and cytoplasmic tail deleted. Among these, the mRNA vaccine lacking the SP failed to induce neutralizing antibodies in the mouse model [146]. The SP plays a crucial role in the expression, secretion, and proper folding of the S protein.

Researchers introduced tPA and IL-6 SPs in the middle of the 5′ Untranslated Region (5′ UTR) and RBD, respectively. In a BALb/c mouse model, this strategy more effectively enhanced the immune response compared to the wild type. Additionally, tPA and IL-6 demonstrated stronger binding to the SRP54M subunit, and this binding ability was positively correlated with antigen translation efficiency [147]. In a study by Yang et al., to enhance the immunogenicity of the monkeypox virus M1R, an SP was added to the extracellular domain of A35R (a homolog of the A33R protein from vaccinia virus) to guide the expression of the fusion protein. This approach boosted immunogenicity and elicited a more effective immune response to neutralize the virus [148]. Proper selection or modification of SPs (such as tPA and IL-6) can elevate antigen presentation levels, thereby inducing a strong immune response at lower mRNA doses.

In addition to the optimization strategies discussed above, several mRNA vaccines have already received regulatory approval and have been deployed globally. During the COVID-19 pandemic, BNT162b2 (Pfizer/BioNTech, New York, NY, USA) and mRNA-1273 (Moderna, Inc., Cambridge, MA, USA) became the first FDA-approved mRNA vaccines, demonstrating over 90% efficacy with strong humoral and cellular responses [150]. Both encode the native SARS-CoV-2 spike protein SP within their ORFs to ensure efficient antigen expression and secretion, enhancing immunogenicity. On 31 May 2024, the FDA approved mRNA-1345 (Moderna, Inc., Cambridge, MA, USA) as the first mRNA vaccine targeting respiratory syncytial virus (RSV) [151]. This vaccine is indicated for the prevention of RSV-associated lower respiratory tract disease in individuals aged 60 years and above. Notably, the SPs employed in these approved mRNA vaccines are derived from endogenous viral antigens, such as the native S protein of SARS-CoV-2, suggesting that SPs originating from self or viral structural proteins may facilitate immune tolerance and efficient protein processing. This observation may inform future SP selection strategies in mRNA vaccine design.

## 5. Concluding Remark

This review primarily discusses the structure, function, and related applications of N-terminal SPs. In addition, there are some secretory proteins with C-terminal SPs, such as peroxisomal targeting signals and nuclear localization signals [44]. A typical peroxisomal targeting signal type 1 (PTS1) consists of a tripeptide sequence Ser-Lys-Leu-COO- at the protein’s carboxyl terminus. This C-terminal SP directs the protein to peroxisomes with high specificity [152,153]. Unlike traditional N-terminal SP, PTS1 does not rely on the Srp pathway. It binds to the TRR domain of peroxisomal factor 5 (PEX5), which depends on a more intricate conformational change. Compared to N-terminal SPs, the concept of C-terminal SPs has been less extensively studied in the literature, with limited related keywords and research depth. This also indirectly suggests that the current tools for predicting SPs have limitations. Most of the SPs we obtain are derived from a limited amount of experimental data. Consequently, there is a need for high-throughput studies on SPs, the construction of SP libraries, and the development of more powerful prediction tools.

SPs, as molecular codes for protein targeting and transport, offer natural insights into protein engineering due to their sequence conservation and functional plasticity. The general strategies for screening and optimizing SPs during protein expression mainly include a. replacing with highly efficient secretory SPs (mouse immunoglobulin IgG2a, tPA, Gaussia luciferase, and immunoglobulin light chain [139,154,155]). b. The modification of the primary structure of the SP: The primary structure of SPs consists of three functional regions, and rational modifications can be made using bioinformatics software like Signal6.0 to predict SPs and analyze functional domains. Generally, increasing the positive charge in the N-terminal region and hydrophobicity in the H-region while removing polar amino acids in the C-terminal region to bring the H-region closer to the cleavage site can generate more efficient modified SPs. c. Host codon optimization: Optimizing host codon preferences is a key factor in improving translation efficiency. Despite codon redundancy, different organisms exhibit different frequencies in the usage of synonymous codons. Selecting codons preferred by the host cell to encode the SP can improve translation efficiency. d. The addition of the Kozak sequence (ACCACCAUGG) before the start codon of the target fragment: The Kozak sequence not only significantly improves protein secretion efficiency but also ensures proper folding and modification of the nascent peptide chain [156].

Although SPs are generally universal across species, studies have shown that the same SP can result in significant differences in protein secretion when used in different hosts [16]. Furthermore, not all secretory proteins are suitable for heterologous SPs. Research has confirmed that when using natural SPs in *L. lactis*, the secretion efficiency is comparable to or even higher than that with heterologous SPs [157]. However, the introduction of SPs does not always guarantee high-efficiency protein secretion. Therefore, optimizing recombinant protein production requires not only the rational design and screening of SPs but also consideration of the specificity of SP enzymes as substrates, as well as a comprehensive understanding of the dynamic process of overall protein secretion and processing.

SPs, originally known for guiding the synthesis and transport of nascent proteins, have evolved to play a multifaceted role in immune regulation. This includes enhancing antigen secretion, assisting in the subcellular localization of target proteins to the ER, promoting MHC I molecule presentation, and subsequently modulating both humoral and cellular immune responses. Research shows that SPs not only impact the immunogenicity of vaccine antigens but also play significant roles in processes such as regulating hormone secretion and mediating viral immune escape. The development of SP vaccines extends beyond the modification of the peptide itself, requiring a synergistic combination of components, such as efficient secretion expression systems, appropriate delivery systems, and optimized adjuvant combinations. Furthermore, circular RNA (circRNA) vaccines, a novel vaccine platform, possess a covalently closed single-strand ring structure, which makes them more stable compared to linear mRNA vaccines [158]. CircRNA vaccines exhibit low immunogenicity and toxicity while maintaining the ability to continuously produce antigens, effectively extending antigen presentation time [159]. Given these advantages, combining SPs with circRNA vaccines may yield synergistic effects, maximizing the efficacy of SP-based vaccines. Currently, most SP vaccines are still in trial and experimental phases. For these candidate vaccines, widespread application will require overcoming various challenges. The effectiveness, long-term protective effects, and safety evaluation of SP-based vaccines still have a long way to go. With further research, SP-based vaccines are expected to make a breakthrough transition from laboratory studies to clinical applications, providing innovative solutions for global public health.

## Figures and Tables

**Table 1:** Characteristics of different transport pathways.

Pathway	Srp	Sec	Tat
Scope	All life groups. More common in eukaryotes	Most Gram-negative bacteria, some Gram-positive bacteria	Archaea, Gram-positive bacteria, plant chloroplasts, and some plant mitochondria
Transported proteins	Mainly transports proteins that remain unmodified in the inner membrane	Unfolded proteins	Completely folded proteins, especially proteins that require co-factor binding for folding and multi-protein complexes
Mechanism	Co-translational translocation	Post-translational translocation	Post-translational translocation
Recognition	In eukaryotes, the SP length and amino acids are highly compatible with ER Sec61; in prokaryotes, it binds FtsY to deliver the complex to the SecYEG translocon	Specific SecB SP	S/T-RRXFLK motif
Energy	GTP	ATP	PMF

**Table 2:** Classical and atypical pathways.

Characteristics	Classical Pathway	Atypical Pathway
SP	Essential	Non-essential
Vesicular transport	Strictly dependent on COPII (from ER to Golgi apparatus), COPI (retrograde transport), and secretory vesicles	Types I and II have no vesicles, partially dependent (Type III: autophagosomes/lysosomes; Type IV: Golgi bypass)
Energy consumption per transport event	High (SRP needs to bind GTP, Sec61 translocation, and vesicular transport)	Relatively low (passive diffusion, ABC transporters require ATP but with low efficiency)
Secretion efficiency	Relatively high efficiency	Relatively low (selective secretion: stress proteins, inflammatory factors)

## Data Availability

No data was used for the research described in the article.
